# Effect of solute atoms on dislocation motion in Mg: An electronic structure perspective

**DOI:** 10.1038/srep08793

**Published:** 2015-03-05

**Authors:** T. Tsuru, D. C. Chrzan

**Affiliations:** 1 Nuclear Science and Engineering Center, Japan Atomic Energy Agency, 2-4 Shirakata-Shirane, Tokai-mura, Ibaraki 319-1195, Japan; 2 Materials Science & Engineering, University of California, Berkeley, California. 94720, USA

## Abstract

Solution strengthening is a well-known approach to tailoring the mechanical properties of structural alloys. Ultimately, the properties of the dislocation/solute interaction are rooted in the electronic structure of the alloy. Accordingly, we compute the electronic structure associated with, and the energy barriers to dislocation cross-slip. The energy barriers so obtained can be used in the development of multiscale models for dislocation mediated plasticity. The computed electronic structure can be used to identify substitutional solutes likely to interact strongly with the dislocation. Using the example of a-type screw dislocations in Mg, we compute accurately the Peierls barrier to prismatic plane slip and argue that Y, Ca, Ti, and Zr should interact strongly with the studied dislocation, and thereby decrease the dislocation slip anisotropy in the alloy.

Magnesium alloys have great potential for the next generation of structural materials. Presently, Mg alloys are the lightest metals in practical use. Widespread application, however, has been limited because of a key weakness stemming from their hexagonal-close-packed (hcp) crystal structure: Their elongation-to-failure is extremely low due to the strong anisotropy in plastic deformation^1^.

A number of studies have focused on improving the ductility of Mg alloys. One approach is to enhance their ductility and strength through grain refinement^2^,^3^,^4^ or controlling spacing of stacking faults^5^. However, the expensive refining process drives up materials costs. Another approach is to introduce solute atoms. Fracture of magnesium alloys generally occurs as a result of strain concentration generated by piled-up basal dislocation at {1012}-type deformation twin, both of which are introduced in the early stage of the plastic deformation of Mg^6^. The atomic structure and crystallographic orientation between basal dislocation and {1012} twin in Mg has become clear^7^,^8^,^9^. The pileup of dislocations arises, in part, because slip on the basal plane is substantially easier than slip on prismatic and pyramidal planes^10^. This failure mechanism might well be alleviated if one can increase the stress required for basal plane slip, while simultaneously enhancing the probability for dislocation cross slip.

Theoretically, it is now clear that the chemistry of dislocation solute interactions can play a large role in the net effect of the solute on twining^11^ and dislocation mobility^12^,^13^,^14^. Recent first-principles calculation with flexible-boundary-conditions allowed for the computation of the interaction energy between a dislocation and solute atoms, and to predict the solution strengthening through an energy-based calculation of the equilibrium structure of the dislocation^15^. The dislocation core structure and the effect of chemical interaction on solid-solution strengthening were predicted^16^,^17^.

Solution softening has a critical role in changing the fracture mode of Mg, and this change is associated with the improved ductility^18^. However, the origins of this softening effect can be difficult to discern within available theories. In a previous report^19^, the softening effect of Y within Mg was studied by combining first-principles electronic structure based calculations of generalized stacking fault energies with the Peierls-Nabarro model^20^,^21^. While these calculations predict the softening effect of Y, the underlying quantum mechanical origins of the effects were not identified. Thus this model is not able to identify easily other candidate alloying additions for increasing the ductility of Mg.

Here, we introduce an electronic structure based approach to understanding the dislocation solute interaction. Rather than focusing solely on the total energy of the dislocation core, and the computation of the Peierls barrier, we additionally focus on the changes in the electronic structure that take place while the straight dislocation is overcoming its Peierls barrier in the elemental metal. These changes in electronic energy levels, as revealed by changes in the electronic density of states (DOS), are compared with DOS projected onto substitutional solutes in the same metal, and the comparison is used to gauge qualitatively the solute/dislocation interaction strength. Though the procedure should be generally applicable, we apply it to solute/dislocation interactions in Mg alloys, revealing clearly the effects of Y on the motion of screw dislocations in Mg. We then use this understanding to identify additional alloying elements likely to have a similar effect on the mobility of these dislocations.

## Results

### Dislocation core structure and motion in pure Mg

The dislocation core structures and Peierls barriers in a periodic supercell^22^,^23^ are considered. An initial atomic configuration is constructed by solving for the displacement field of the dislocation dipole within a periodic continuum linear elasticity theory (see Methods and Supplementary Information). Within this method, the distortion tensor arising from the dislocations is assumed to be periodic, and is expanded in a Fourier series following reference [23]. The Fourier coefficients for the distortion tensor are computed by minimizing the total elastic energy of the system subject to the constraints placed on the distortion tensor by the presence of the dislocations within the unit cell. The atomic displacements are then found by integrating the distortion tensor.

**Figure 1:**
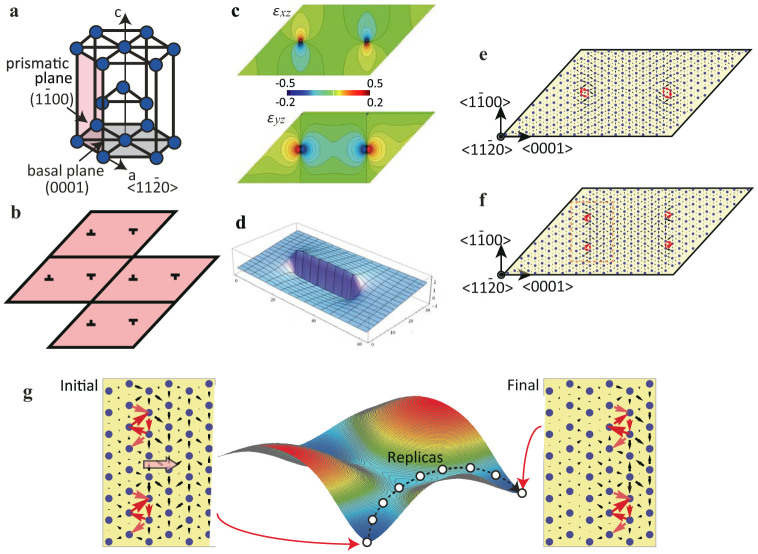
Construction of dislocation core structure of hcp crystals. (a) A unit cell of hcp structure with basal and prismatic plane identified. (b) Periodic array of dislocation dipoles. (c) Strain fields of *ε_xz_* and *ε_yz_* calculated by the elastic theory in a periodic cell. (d) Displacement field of *u_z_* component obtained by the path integration of strain field along *x* and *y*. (e) The differential displacements map of dislocation core predicted by the elasticity theory described in the text. (f) Same as e, but map of the relaxed dislocation core by DFT. (g) Schematic image of dislocation moving along to the 〈0001〉 direction.

The initial conditions for the density functional theory computations assume that each cell contains an a-type screw dislocation dipole composed of dislocations with Burgers vectors of 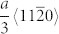 on the basal or prismatic plane (Fig. 1a), where a and c are defined as the lattice constants of Mg (Supplementary Table 1S). The structure of the periodic array is shown in Fig. 1b. The (periodic) strain fields of the periodic array of screw dislocation dipoles are shown in Fig. 1c. Though the strain fields are periodic, the predicted displacements in the z-direction (the line direction of the dislocations) are not (Fig. 1d). The elasticity theory prediction for the atomic displacements within the unit cell is shown in Fig. 1e using the differential displacement map introduced by Vitek^24^,^25^. The relative displacement associated with first to third nearest neighbor atoms in 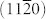 plane with in-plane cutoff distance 
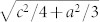
 is visualized by vector arrows. Note that this initial solution assumes that the dislocations do not dissociate into partials. This unit cell is then relaxed using DFT (see Supplementary Information for details) and the resulting structure is shown in Fig. 1f. After the relaxation, the screw dislocations display the expected dissociation on the basal planes.

**Figure 2:**
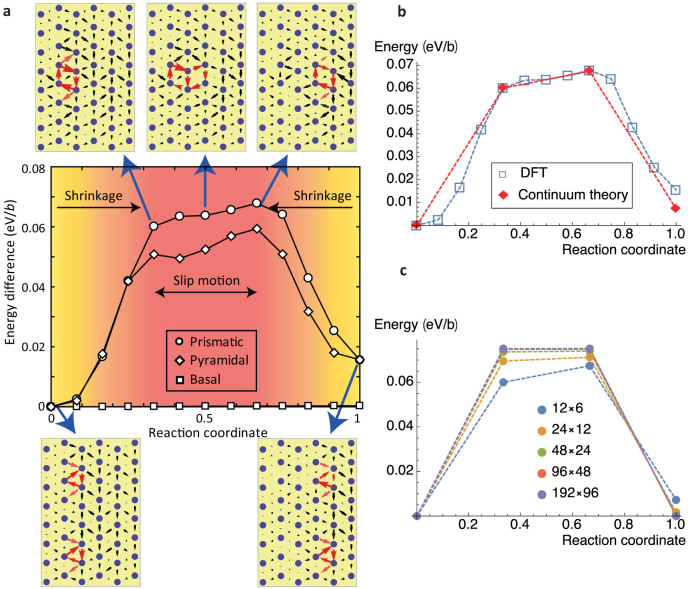
Energy difference during the dislocation motion. (a) The stable energy paths are calculated by NEB simulations for the motion of screw dislocation in the basal, prismatic and pyramidal plane. Some important transition states for motion on the prismatic plane are visualized by the differential displacement map. Reaction coordinate is divided into three parts depending on the type of event; the constriction of partial dislocations to create a compact core, the motion of compact core, and the redissociation of the dislocation into partials. (b) The energy difference in the prismatic plane compared with fitted continuum theory prediction. (c) The effect of supercell size on the energy difference in the process of dislocation motion within the continuum theory. Based on this continuum extension of the atomic scale results, the Peierls barrier of an isolated dislocation gliding on the prismatic plane is 0.075 eV/*b*.

We used the nudged elastic band (NEB) method^26^ to compute the physical and electronic structure of the dislocation as it passes over the Peierls barrier. Two initial configurations corresponding to the most stable dislocation dipole structure position and the position where one of the dislocations moves along the 〈0001〉 direction were prepared, as shown schematically in Fig. 1g. (The initial and final configurations of the NEB calculation are also represented schematically in Figure S2 configurations (1) and (4) respectively.) The NEB calculations were performed with 11 relaxing replicas. The energy difference during dislocation glide on basal, prismatic and pyramidal plane is shown in Fig. 2 as well as the differential displacements at several important transition states for the case of glide on the prismatic plane. It is seen from the initial and final states that this screw dislocation dissociates on the basal plane into partial dislocations. This is consistent with the results of previous theoretical studies^16^,^17^,^19^, and arises from the low energy of the basal plane stacking fault. The dislocations glide easily on the basal plane – there is essentially no energy barrier between symmetrically equivalent structures. On the other hand, the motion on the prismatic plane requires surmounting an energy barrier as demonstrated by the NEB calculation.

Dislocation motion on the prismatic plane can be divided into three steps, an observation consistent with the Friedel-Escaig mechanism proposed by Couret and Caillard^10^. The first step is the constriction of the partial dislocations on the basal plane that yields the compact core screw dislocation capable of gliding on the prismatic plane. The second step is glide on the prismatic plane. The third step is redissociation into partials split on the basal plane. From Fig. 2 it is evident that a major portion of the energy barrier for motion of the dislocation on the prismatic plane is the energy required to constrict the partial dislocations enabling cross-slip. This energy accounts for most of the (approximately) 0.06 eV/*b* Peierls barrier, with *b* the Burgers vector of the dislocation. The apparent barrier for glide of the compact dislocation is much lower approximately 0.01 eV/*b* for prismatic and pyramidal glide.

The energy barrier so computed includes two general contributions: (I) the energy barrier expected for an isolated dislocation and (II) the changes to this barrier arising from the interaction with the periodic images. This second contribution can rightly be considered an artifact of the supercell geometry, and so we need a means to understand its impact. The calculation of the Peierls barrier within a periodic supercell approach has been considered by Cai et al.^27^ and Pizzagalli et al.^28^ Here, we again choose to adapt Daw's formulation of the periodic elasticity problem^23^ to model the changes in elastic energy within our periodic supercell as the dislocation moves over the Peierls barrier (Supplementary Table S2 and Fig. S2).

Specifically, we note that the motion of the dislocation on the prismatic plane can be modeled at the continuum scale by accounting for changes in the elastic energy and the stacking fault energy upon constriction of the partials to form a compact core dislocation. Within Daw's periodic supercell formulation, we can compute the changes in elastic energy associated with constricting the partials to within twice the core distance, *r_c_*, (at this distance the dislocation core regions just begin to overlap) moving the compact dislocation by one lattice parameter in the slip direction, and then re-dissociating the compact core into partials. The changes in elastic energy so computed are then combined with the computed stacking fault energy to predict the change in total energy associated with moving the dislocation over its Peierls barrier. The value of *r_c_* is chosen so as to best fit the DFT results (see Supplementary Information for details). This procedure leads to the choice *r_c_* = 0.9*b*.

The comparison between the continuum approach and the atomic scale results is plotted in Fig. 2b. The continuum theory does very well overall, only showing a slight difference at the endpoint (that can be attributed to a small change in dislocation core energy – see Supplementary Table S2 and Fig. S2). The implication is that the bulk of the Peierls barrier to prismatic glide is, in fact, the increase in elastic energy and the associated reduction in stacking fault energy associated with the transformation to a compact core. Moreover, it is clear that the classical model for this constriction process is a robust and accurate description, now verified by a calculation rooted firmly in quantum mechanics.

The linear elastic description of the periodic supercell enables one to discern the effects of the periodicity on the computed Peierls barrier. Figure 2c shows the continuum theory predictions for the energy barrier as a function of supercell size. The predicted Peierls barrier converges to 0.075 eV/*b* for a supercell containing 96 × 48 four-atom unit cells. Based on this calculation, the primary effect of the supercell geometry is to reduce the partial constriction energy by approximately 0.015 eV/*b*. A secondary effect is to introduce an additional barrier to dislocation motion, 0.007 eV/*b*. Both effects can be eliminated by considering the continuum theory in the large supercell limit (~18,000 atoms).

**Figure 3:**
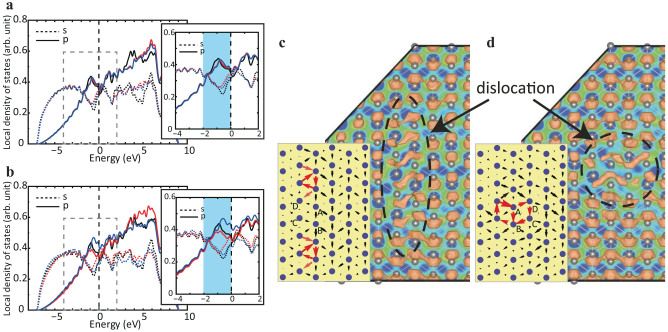
Electronic structure of partial and perfect dislocation. (a) Site-projected DOS for different locations of atoms within a dislocation core and in bulk Mg. Atoms marked with A and C exhibit the same electronic structure, indicated by red lines. The same is true for B and D, and their projected DOS are indicated by blue line. The black lines indicate the projected DOS for a bulk Mg atom. *s*-states are depicted by dotted lines, and *p*-states are shown with solid lines. The Fermi energy is chosen as the zero of energy. Energy states with respect to a specific energy range from −4.0 to 2.0 eV are shown in the inset. (b) Same as a, but for the compact dislocation. Note that the compact core shows substantial differences from the bulk atoms in their projected DOS near the Fermi level. (c) Differential displacement map and overview of partial charge density for a partial dislocation with respect to a specific energy range (−2.0 to 0 eV). (d) same as c, but for a perfect dislocation.

Returning to the quantum mechanical formulation of the problem, we note that it allows us to understand the changes in the electronic structure associated with motion of the dislocation along the prismatic plane. The electronic structures of the initial configuration and the sixth replica corresponding to the partial and compact dislocations, respectively, are shown in Fig. 3. We examined the site-projected DOS on four atom positions, labelled A-D, and an additional atom far from the dislocation core. Thus, we are able to track the evolution of the site-projected DOS as the dislocation passes over the Peierls barrier.

Examination of the DOS presented in Fig. 3 establishes two points. First, dislocations do not bring great changes in the states of the outermost *s* electrons of Mg. Second, the *p*-states just below the Fermi level are influenced strongly by the passage of a dislocation. While the projected DOS for the atoms A and C, indicated by red lines, is reduced by the compact dislocation, the projected DOS for atoms b and d are increased. Further, while there are small changes associated with the site-projected DOS of these atoms in the dissociated dislocation core, the rearrangements are much more pronounced in the case of the compact core. These *p*-states, therefore, participate in the bonding rearrangements that take place as the dislocation transitions from dissociated partials to a compact dislocation in order to surmount its Peierls barrier. A similar effect is apparent in the charge density derived from the states near the Fermi level. Figures. 3c–d show the charge density plots for the dissociated and compact dislocations, which are drawn by VESTA software^29^. There is substantial rearrangement of the charge upon the transition from dissociated to compact core.

These changes in the electronic structure of the dislocation as it surmounts its Peierls barrier constitute, in some sense, the electronic structure of the dislocation motion. We are now in the position to identify substitutional elements that might interact strongly with the dislocations in Mg.

### Electronic states of solutes in Mg

The principle underlying our analysis is very simple. Introduction of a substitutional solute atom changes the electronic structure near the solute. If these changes occur in the same energy range associated with the changes in the electronic structure of the dislocation during motion, strong chemical hybridization can occur between the solute atom states and those states associated with dislocation motion. If these states also lie below the Fermi level, this hybridization will lead to a strong physical interaction between the dislocation and the substitutional solute atom.

**Figure 4:**
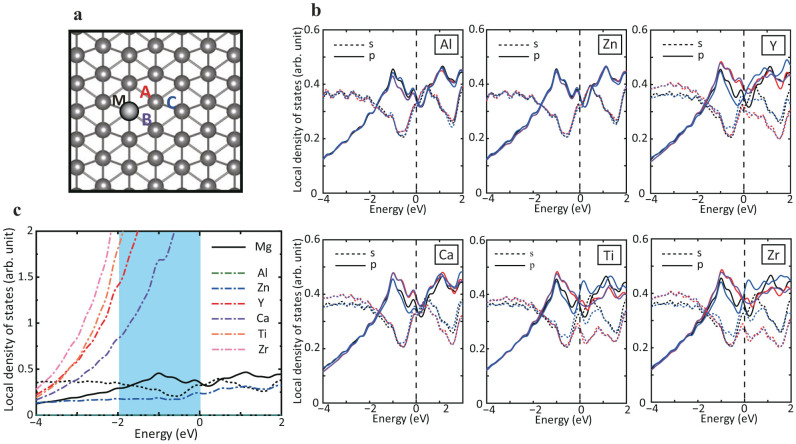
Electronic structure around a solute atom. (a) Atomic configuration of hcp Mg with a solute atom. (b) Projected density of states for three atoms around a solute in perfect crystal. The color codes are corresponding to atoms A, B and C in Fig. 4a: Red, purple and blue lines indicate A, B and C, respectively, and curves for defect free region is drawn by black lines. Both *s* and *p* states are depicted by dot and solid lines, respectively. (c) Projected density of states for solute elements (Al, Zn, Y, Ca, Ti and Zr) in perfect crystal.

To explore this idea, we computed the electronic structures of Al, Zn, Y, Ca, Ti and Zr substitutional atoms within Mg (see Computational Details). The site-projected DOS are shown in Fig. 4. From the figure, it is clear that Al and Zn have only minimal effect on the DOS in the energy range associated with the motion of the dislocation (−2 eV to 0 eV). In contrast, Y, Ca, Ti, and Zr show relatively large changes in the electronic states in this energy range. We expect, therefore, that substitutional Al and Zn will not interact strongly with the dislocation, but that substitutional Y, Ca, Ti and Zr will.

### Effect of solutes on dislocation core

**Figure 5:**
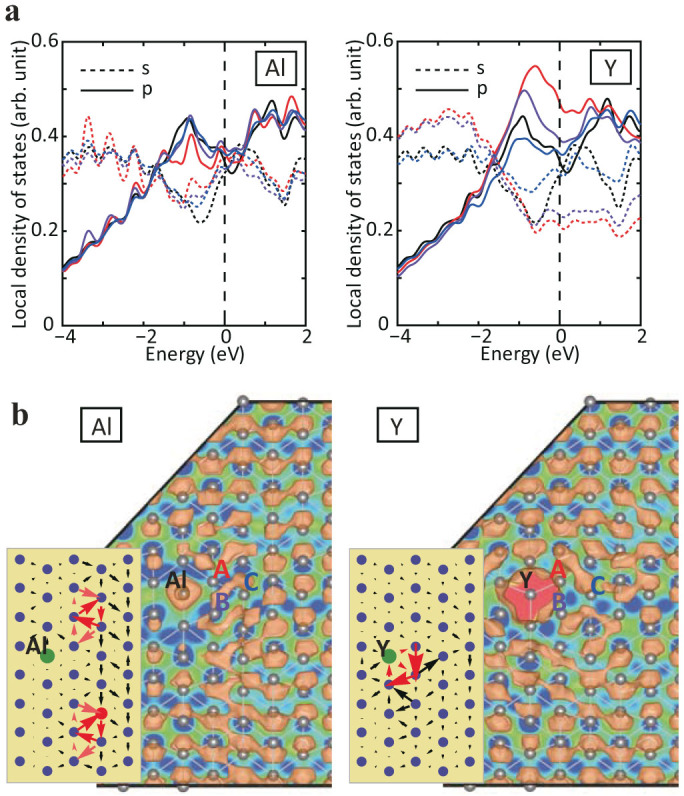
Electronic structure of dislocation core around solution. (a) Projected density of states for three atoms constituting dislocation core as well as the perfect crystal. The color codes are corresponding to atoms A, B and C in Fig. 5b: Red, purple and blue lines indicate A, B and C, respectively, and curves for defect free region is drawn by black lines. Both *s* and *p* states are depicted by dot and solid lines, respectively. (b) Differential displacement map and partial charge density for the dislocation core with a solution with respect to a specific energy range (−2.0 to 0 eV).

Based on this evidence, we then considered directly the interaction of the dislocation with substitutional solute atoms. Specifically, we confirmed that the dislocation core structure is strongly influenced by Y, Ca, Ti and Zr, and is relatively unchanged in the case of Al and Zn. A typical comparison is shown in Fig. 5. The dislocation remains dissociated into partial dislocations in case that the Al and Zn solutes are added, because Al solute does not influence *p* states of atoms near the dislocation core. In contrast, it is seen that a dislocation dissociated in the basal plane shrinks immediately as a result of the strong interaction with Y, Ca, Ti and Zr solutions. It is interesting to note that the partial charge density for a dislocation core with Al and Y shows a similar distribution to those for a partial and perfect dislocation cores in pure Mg, respectively. The density of states of these solute elements added near the dislocation core of Mg are shown in Fig. 4b, where *s* and *p* states of Mg and *d* states of each solute element are given in the same graph. A clear distinction can be seen between the two types of groups: Al does not have *d* states and Zn has few *d* states if any in the energy range of interest. On the other hand, Y, Ca, Ti and Zr show prominent *d* states between −2.0 and 0.0 eV. These *d*-states are available for hybridization with the *p*-states of the Mg, and ultimately lead to the strong interaction between these alloying additions and the dislocation core structure. These calculations, therefore, confirm that one can use the electronic structure of the dislocation motion to identify substitutional alloying elements that interact strongly with the dislocations in Mg. However, we need to understand the effects of this interaction on dislocation motion.

**Figure 6:**
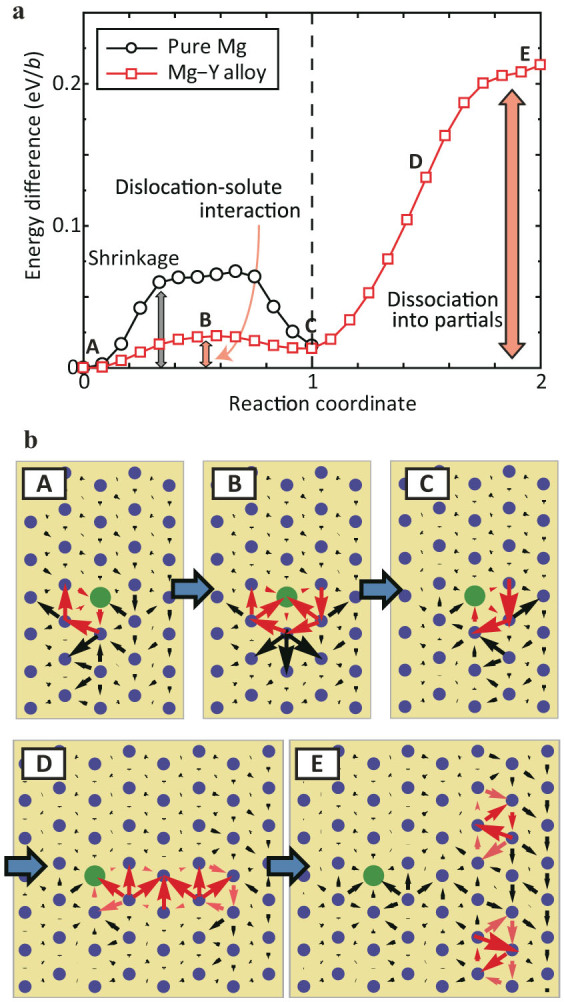
Effect of solid solution on softening and hardening. (a) Energy differences during the dislocation motion in prismatic plane for pure Mg and Mg-Y system. The curve of pure Mg drawn by black line is the same as Fig. 2. Energy curve when dislocation pass through Y solution is indicated by red line. (b) Differential displacement maps in the process that dislocation overcomes and moves away from the Y solution.

As shown above, in pure Mg glide of a screw dislocation on the prismatic plane is only possible in the compact core configuration. Our calculations show that the strongly interacting solutes bind to, and thus stabilize, the compact core. To explore the range of this binding, we performed a NEB calculation of the motion of dislocation in a cell with one column of Mg atoms replaced by Y. The results of this calculation are shown in Fig. 6.

There are three important observations that arise from study of Fig. 6. First, the presence of the Y leads to a core transformation from dissociated core to a compact core, with the Y atoms within the core. Second, the compact core is stable for a broad range of distances between it and the Y, approximately 3/2 *c* in either direction, with *c* the length of the high symmetry axis in Mg. Third, the dislocation core is bound to the row of Y atoms by at least 0.2 eV/*b*, and this energy increase takes place (primarily) over a distance of 3/2 *c*.

We are now in the position to consider the effects of alloying additions on the slip anisotropy of Mg. As stated above, slip anisotropy is one of the origins of the poor ductility in Mg. In the absence of solute atoms, screw dislocations glide on the basal plane with almost no Peierls barrier, where as the Peierls barrier for prismatic and pyramidal slip are quite large, and this leads to elastic limit ratio for slip on these planes^10^: 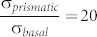. Given this situation, slip anisotropy can be improved if one increases the strength of basal plane slip.

### Hardening effect of solutes of basal plane

Consider the basal plane. Introduction of Y as substitutional solute will lead to, locally, strong binding of the basal plane screw dislocation to the Y atom. If one assumes that the final state of the NEB calculation presented in Fig. 6 is not interacting with the dislocation, one expects this binding energy to be approximately 0.2 eV per Y atom. As noted in Fig. 6, this binding is dissipated over a distance of roughly 3/2 *c*. Using the binding energy per unit length, and the estimate of the interaction distance, one can estimate the pinning strength of an assumed immobile single Y atom^30^,^31^,^32^ (this calculation is presented in the Supplementary Information.). Incorporating this pinning strength into simple models for obstacle controlled glide, we estimate that Y levels of 1 at.-% will increase the yield stress of the basal glide plane by approximately 90 MPa. If the strengthening of the prismatic slip plane is similar, in magnitude, the elastic limit ratio is reduced to 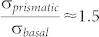. The introduction of 1 at.-% Y is expected to reduce the elastic limit ratio for slip on these two planes by a factor greater than 10. However, the strengthening of the prismatic plane is likely to be reduced (as discussed below) suggesting that Y may be even more effective at reducing slip anisotropy.

Consider now glide of the same screw dislocation on the prismatic plane. As noted above, the largest contribution to the Peierls barrier for slip on this plane is the energy associated forming the compact core from the dissociated partials. However, in the presence of a Y atom, the core does not dissociate. In fact, motion of the dislocation through the Y atom requires overcoming only a small Peierls barrier, approximately 0.02 eV/*b*. (We do not expect this barrier to be subject to large corrections from the periodic supercell approach.) So, if the density of Y atoms can be made sufficiently large to keep large portions of the screw dislocation cores compact, cross slip onto, and glide on the prismatic plane will require substantially less stress.

We can estimate the density of solute atoms necessary to maintain the compact core structure for the screw dislocations in Mg using the data in Fig. 6. If we assume that the extent of the solute atom's electronic structure along the dislocation core is roughly the same as its extent normal to the core, we expect that a single solute atom influenced dislocation properties within a spherical region of the crystal with volume 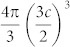. This volume contains, within pure Mg, approximately 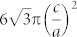 atoms. Again, at approximately 1 at.-% Y, large portions of the dislocation core are expected to be found in the compact configuration.

Experimentally, there is some support for our model. Recent experimental studies have indeed found that Ca solutes enhance non-basal slip deformation (H. Somekawa, private communication) although they make the fracture toughness weaker as a result from the grain boundary segregation^30^, and that Zr solutes improve the elongation to failure with a positive effect on grain refinement^31^,^32^ in which multiple slip plays an important role.

## Discussion

There are three primary conclusions from our work. First we have shown that by combining a continuum linear elasticity theory for periodic dislocation arrays with density functional theory calculations employing the nudged-elastic-band formalism for finding saddle point, that we can compute the Peierls barrier for 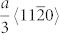 screw dislocation slip on the prismatic planes of Mg. For an isolated dislocation, this barrier is computed to be 0.075 eV/*b*. Moreover, the barrier arises from the constriction energy of the partial dislocations, exactly as expected within the Friedel-Escaig model for cross-slip.

Second, we have computed the changes in electronic structure associated with the motion of the dislocation over its Peierls barrier. For the 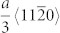 screw dislocation in Mg, these changes occur primarily in p-states right below the Fermi level. Alloying additions with electronic states in this energy range are, therefore, likely to interact strongly with the dislocation core, and thereby influence its motion. These additions, in turn, can be identified through simple computation of their electronic structure when embedded within an otherwise perfect bulk. Based on this analysis, we argue that substitutional Y, Ca, Ti and Zr are expected to interact strongly with the screw dislocation cores in Mg, whereas Zn and Al are expected to have little interaction.

Third, we have shown by direct computation that Y stabilizes the compact core for the 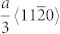 screw dislocations in Mg. This compact core is then able to glide on the basal, prismatic and pyramidal planes. So the same solute addition both increases the stress required for slip on the basal plane, and enables cross-slip to prismatic and pyramidal planes. Both of these effects have the potential to increase the ductility of Mg, through the blunting of basal plane dislocation pileups that are linked to fracture.

## Methods

For more details, see Supplementary Information.

### Dislocation in a periodic cell

The introduction of dislocations within our periodic is accomplished through application of a continuum linear elastic theory solution for the periodic dislocation dipole array^23^. The distortion field caused by periodic distribution of dislocations is expressed as a Fourier series:
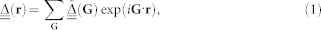
with Δ = (**r**) the distortion tensor at point **r**, **G** the reciprocal lattice vectors, and 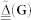 the Fourier coefficients describing the distortion tensor. The elastic energy per unit length of the supercell can be expressed in terms the 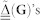:
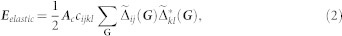
with *A_c_* the area of the supercell containing the dislocation dipole and *c_ijkl_* the elastic constants of the material. The distortion field is then chosen to minimize the total elastic energy subject to the topological constraints imposed by the dislocations. The predicted displacement at the atomic positions is obtained by a line integral starting from a reference coordinate. In this work, the screw dislocation dipole with Burgers vectors of the type 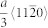 is studied. The dipole is inserted into a 288 atom supercell with the dimension of 12 × 6 periodic units along [0001] and 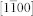, respectively (Supplementary Fig. S1).

### Computational details

DFT calculations were carried out using the Vienna Ab initio simulation package (VASP)^33^,^34^ with the Perdew–Burke–Ernzerhof formulation of the generalized gradient approximation to the exchange-correlation density functional^35^. The Brillouin-zone *k*-point samplings were chosen using the Monkhorst–Pack algorithm^36^. The plane-wave energy cutoff was set at 400 eV. The outer *s* and semi-core *p* electrons were considered as valence electrons. 1 × 1 × 9 and 2 × 2 × 25 *k*-point samplings were used for structural relaxation and density of states calculation, respectively. The fully relaxed configurations were obtained by the conjugate gradient method that terminated the search when force on all the atoms was reduced to less than 0.005 eV/Å. The transition state during dislocation motion were calculated using NEB calculations^26^ with eleven intermediate replica images, and forces converged to better than 0.01 eV/Å.

## Author Contributions

T.T. and D.C.C. designed the research and established the theoretical approach, and performed elasticity theory calculations. T.T. carried out the DFT calculations. T.T. and D.C.C. interpreted the results and prepared the manuscript.

## Supplementary Material

Supplementary Information Effect of solute atoms on dislocation motion in Mg: An electronic structure perspective
