# Supplementary material for: Effect of solute atoms on dislocation motion in Mg: An electronic structure perspective
Source: Sci Rep. 2015 Mar 5;5:8793. doi: 10.1038/srep08793 (PMC4350096; doi:10.1038/srep08793)
Supplement: Supplementary Information — Effect of solute atoms on dislocation motion in Mg: An electronic structure perspective [file srep08793-s1.doc]

SUPPLEMENTARY INFORMATION

Effect of solute atoms on dislocation motion in Mg: An electronic structure perspective

Tsuru, T.^1,2^ & Chrzan, D. C.^2 *^

^1^ Nuclear Science and Engineering Center, Japan Atomic Energy Agency, 2-4 Shirakata-Shirane, Tokai-mura, Ibaraki 319-1195, Japan

^2^ Material Science & Engineering, University of California, Berkeley, California, 94720, USA

^*^­To whom correspondence should be addressed; E-mail: [tsuru.tomohito@jaea.go.jp](mailto:tsuru.tomohito@jaea.go.jp) and ^#^dcchrzan@berkeley.edu

**1. Displacement field of a periodic array of dislocation dipoles**

The periodic dislocation dipole configuration places the dislocations in close proximity. It is, therefore, necessary to have a detailed understanding of the nature of these elastic interactions, and to reflect these elastic interactions within the analysis of the atomic scale results. Here, we briefly describe the linear continuum elasticity theory solution for the periodic dislocation dipole array, and describe how it is used to both provide the initial conditions for our calculations, and to interpret the nudged elastic band (NEB)^1^ calculations for dislocation Peierls barriers.

The elastic field of a dislocation dipole in a periodic cell is solved algebraically using Daw’s approach^2^. The distortion field caused by the periodic distribution of dislocations is defined as the gradient of the displacement field and expressed by a series of plane waves $\underline{\underline{\Delta}}(\mathbf{r})=\sum_{\mathbf{G}} \tilde{\underline{\underline{\Delta}}}(\mathbf{G})\exp(i\mathbf{G}\cdot\mathbf{r})$ with $\mathbf{G}$ the reciprocal lattice vectors corresponding to the chosen supercell periodicity. The elastic energy is related to the distortion through the following equation: $E_{elastic}=\frac{1}{2}A_{c}c_{ijkl}\sum_{\mathbf{G}} \Delta_{ij}\left(\mathbf{G}\right)\Delta_{kl}^{*}\left(\mathbf{G}\right)$, with $A_{c}$ the area of the supercell, and $c_{ijkl}$ the elastic constants of the material in question. In practice, one introduces a core radius, $r_{c}$ , to insure convergence of the summation for the elastic energy. In our work, we have chosen this core radius to be $a/8$for computing the initial displacement field. (We discuss below the choice of $r_{c}$ for elastic energy computations.)

The predicted distortion is found by minimizing the elastic energy of the system subject to the topological constraints imposed on the distortion by the presence of the dislocations^2^. The initial displacements of the atoms are then obtained through a line integral of the distortion beginning at a specified reference point.

The elastic constants and lattice parameters for Mg are obtained using density functional theory (DFT). Our computed values are shown in Table S1.

Table S1. Lattice parameters and elastic constants in hexagonal Mg.

|  | *a* (Å) | *c*/*a* | *C*_11_ (GPa) | *C*_33_ (GPa) | *C*_12_ (GPa) | *C*_13_ (GPa) | *C*_44_ (GPa) |
| --- | --- | --- | --- | --- | --- | --- | --- |
| Present work | 3.203 | 1.614 | 62.4 | 70.9 | 22.0 | 21.9 | 11.7 |
| Exp. ^3^ | 3.209 | 1.623 | 59.3 | 61.5 | 25.7 | 21.4 | 16.4 |
| Other calc. ^4^ | 3.196 | 1.626 | 60.8 | 65.4 | 31.4 | 21.0 | 15.3 |

In this work, screw dislocation dipoles with Burgers vector of the type $\frac{a}{3}\left\langle11\bar{2}0\right\rangle$ are considered. A dislocation dipole is inserted into a supercell with the dimension of 12 × 6 unit cells along $\left\langle0001\right\rangle$ and $\left\langle1\bar{1}00\right\rangle$directions, respectively. The (2-D, for this problem) reciprocal lattice vectors can be expressed in terms of the primitive reciprocal lattice vectors, defined to be $\mathbf{a}^{*}$ and $\mathbf{b}^{*}$, as $\mathbf{G}_{hk}=h \mathbf{a}^{*}+k \mathbf{b}^{*}$ with $h,k\in Integers$. Figure S1 **a** plots the predictions of one component of the distortion tensor as a function of the number of $\mathbf{G}$ vectors in the sum. We find that the Fourier series is completely converged (given our choice of core radius *r*_c_ = *b* for this plot) if one chooses both *h* and *k* to range from -20 to 20.

For the predictions of the displacements based on the elasticity theory solution, one must consider several possible dislocation positions, as shown in Fig. S1**b**. The most convenient of these is position No.1 (and symmetrically equivalent positions). This position was used for the initial atomic configuration for the DFT calculations.

The elastic energy of the periodic array of dislocation dipoles depends on the stacking of the unit cells. We considered two possible stackings (Fig. S1 **c**): a dipole stacking, in which the dislocations are arranged to form low angle tilt boundaries, and a quadrupolar stacking, wherein dislocations of opposite sign are stacked upon one another.

The computed elastic energies per Burgers vector are plotted as a function of the spacing between dislocation cores in Fig. S1 **d**. We note that the quadrupolar cell shows the smaller change in elastic energy with changes in dislocation separation. We therefore use the quadrupolar configuration in our analysis.

The introduction of dislocation dipoles within each supercell results in a distortion of the supercell vectors, as noted by Lehto and Öberg^5^. The supercell lattice vectors are, therefore, adjusted accordingly, to reflect a dipole composed of compact dislocations separated by one half the dimension of the cell along the prismatic slip plane.

In the computations that follow, the motion of the dislocations changes the distortion of the supercell vectors, and our computations should reflect this change. However, we found that including these small changes within the electronic structure calculations introduced substantial noise. Specifically, the change in supercell vectors leads to a different sampling of **k**-space in the necessary integrations. We found that this different sampling can reduce the “cancellation of errors” that one gains by sampling **k**-space consistently through computation of the Peierls barrier. We therefore chose to freeze the supercell vectors distorted as described above, and then to correct our final calculations to account for the stress imposed on the dislocations by not allowing relaxation of the supercell lattice vectors. This procedure proved remarkably robust and efficient.

As noted in the main text, we can use a combination of the elasticity theory described above, and a computation of the stacking fault energy to describe the main energetic contributions to the Peierls barrier. (We refer to this as the continuum theory.) More specifically, we model the process as transitions between four states (Fig. S2 **a**): (1) the dissociated partials, (2) constriction of the partials to form a compact dislocation that can glide on the prismatic plane, (3) glide of that dislocation to an adjacent (nominally) symmetrically equivalent position, and (4) redissociation of the dislocation into partials. In considering the constriction of the partials, we need to define a distance at which the partials are considered to form a compact core. We choose this distance to be $2r_{c}$ , where $r_{c}$ is the dislocation core radius employed in the elasticity theory computations. The energy of state (2) is then computed by considering the change in elastic energy arising from the constriction of the partials, and subtracting the stacking fault energy that is lost during the constriction. The energy of state (3) is the energy change associated with moving the contracted partial by one lattice spacing towards the other dislocation in the cell (also dissociated), and accounting for the strain energy associated with not distorting the supercell lattice vectors. The energy for state (4) includes the change in elastic energy associated with the redissociation of the partials, the strain energy associated with holding the supercell lattice vectors rigid, and adding the additional stacking fault energy.

We computed the relevant stacking fault energy of Mg, and found that it is $\gamma=0.001846$ eV/Å^2^. The uniform elastic strain (per supercell) associated with assuming the supercell lattice vectors rigid during the dislocation motion is computed, using the elastic constants reported above, to be 0.02431 eV for the $12\times6$ supercell. Table S2 displays the computed elastic energy contributions arising from the dislocation arrays as a function of supercell size.

By adjusting the value of $r_{c}$used in these calculations, one can fit to the DFT-NEB results. This fitting resulted in $r_{c}=0.9 b$, and leads to the results shown in Fig. 2**b** of the main text. We note that the predictions from the periodic elasticity theory and the DFT-NEB results agree very well – the only noticeable difference is for state (4) where the continuum theory underestimates the final state energy by approximately 0.01 eV/*b*. This difference, we believe, can be attributed to distortions in the dislocation dissociation distance/core structures associated with the change in stress state arising from the change in the periodicity of the dislocation array. These changes lead to, roughly, 0.005 eV/*b* per compact dislocation, a small discrepancy.

As described in the text, the continuum theory is then used to extrapolate the DFT-NEB results to larger supercells. We simply double the size of the cell, fixing $r_{c}$ and the dissociation distance, until the energies for the three states no longer change. The effect of supercell size on the Peierls barrier (as represented by the energy difference between states (1) and (3)) is plotted in Fig. S2**b**. Figure S2**c** plots the energy difference between states (3) and (2). We can see that a supercell at least 96×48 , that would include 18,432 atoms, is necessary to converge the elastic energy contributions to the Peierls barrier.

**Table S2. The elastic energies computed for the four states as a function of supercell dimensions.**

| supercell dimensions | *E*_1_ (eV/Å) | *E*_2_ (eV/Å) | *E*_3_ (eV/Å) | *E*_4_ (eV/Å) |
| --- | --- | --- | --- | --- |
| 12 × 6 | 0.797892 | 0.823968 | 0.818691 | 0.792559 |
| 24 × 12 | 1.136424 | 1.165476 | 1.16411 | 1.135059 |
| 48 × 24 | 1.48084 | 1.511168 | 1.510823 | 1.480496 |
| 96 × 48 | 1.827735 | 1.858425 | 1.858338 | 1.827648 |
| 192 × 96 | 2.175329 | 2.206113 | 2.206092 | 2.175308 |

The results of this procedure are given in Fig. 2**c** of the main text. This procedure enables us to conclude that the Peierls barrier for the motion of an isolated dislocation in Mg (to the extent that a dislocation can be “isolated”) is 0.075 eV/*b*. We also note that the continuum theory predicts no Peierls barrier for a dislocation that remains compact throughout its motion. We, therefore, do not expect the predictions for the motion of the compact dislocation (first part of motion considered in Fig. 6 of the main text) to be much altered by the periodicity of the dipole array.


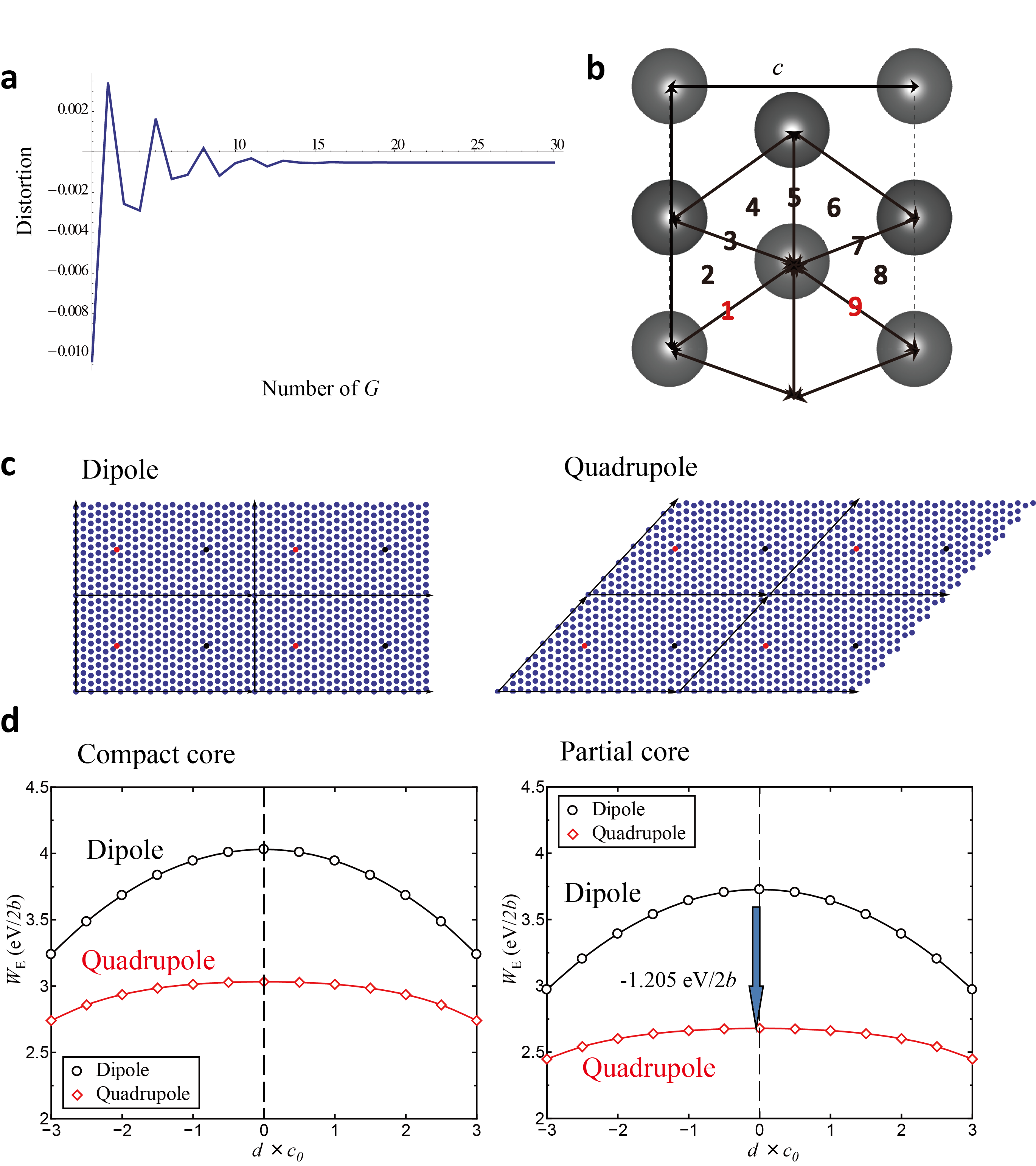


**Figure S1 | Elastic solution of dislocation quadrupole in periodic cell. a, A typical** relationship between the distortion at a point in real space and the number of **G** included in the sum vectors to check convergence (here, *r*_c_ = *b*). **b**, Core positions used to determine energetically preferable configurations. **c**, Two initial configurations of the dislocations showing dipolar and quadrupolar stackings. **d**, Relationship between elastic energy and spacing between dislocation cores in the *c*-direction, where energy difference between dipole and quadrupole for both compact and partial dislocation cores are compared.


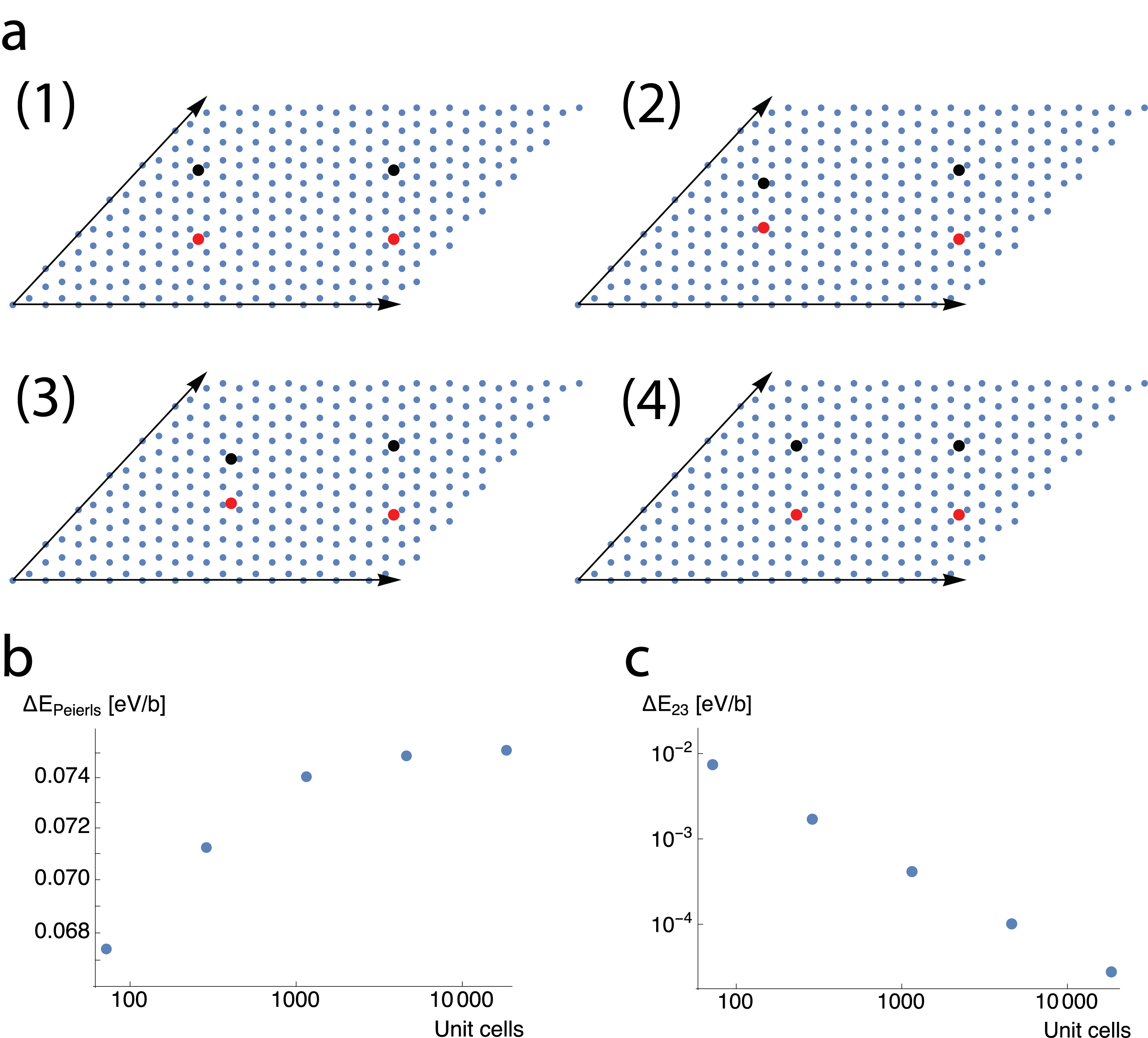


**Figure S2 | Configurations and convergence for continuum theory**. **a**, The four states used in computing the Peierls barrier using the continuum theory. The red and black dots represent the positions of partial dislocations. **b**, A log-log plot of continuum theory prediction for the Peierls barrier as a function of system size. For the purposes of this plot, $\Delta E_{Peierls}\equiv E_{3}-E_{1}$ , where $E_{j}$ is the total energy (elastic plus stacking fault) of state *j*. **c**. A log-log plot of the energy difference between states 2 and 3, $\Delta E_{23}\equiv E_{3}-E_{2}$. As the system size increases, $\Delta E_{23}$ decreases. This suggests that the energy barrier associated with moving the compact dislocation in the DFT-NEB calculations arises, at least in part, from the elastic interactions between the dislocations in the periodic array.

**2. DFT calculation for dislocation quadrupole**

The displacements corresponding to each lattice point, obtained using the continuum linear elasticity theory outlined above, were applied to the atomic model for a dislocation dipole in the quadrupolar stacking configuration. In addition, the periodic supercell vectors were adjusted as discussed above. The dislocation core structure so obtained is used as the initial configuration for DFT calculations.

In this work, density functional theory (DFT) calculations were carried out using the Vienna Ab initio simulation package (VASP)^6,7^ with the Perdew–Burke–Ernzerhof generalized gradient approximation exchange-correlation density functional^8^. The Brillouin-zone *k*-point samplings were chosen using the Monkhorst–Pack algorithm^9^. The plane-wave energy cutoff was set at 400 eV. The outer *s* and semi-core *p* electrons were considered as valence electrons. The fully relaxed configurations were obtained by the conjugate gradient (CG) method when the energy norm of all the atoms converged to better than 0.005 eV/Å. The metastable configuration corresponding to the perfect dislocation was obtained after only 13 CG steps, that is, the elastic solution provides the approximate atomic configuration only if the dislocations do not dissociate.

However, the stable configuration includes dissociated dislocations as shown in Fig. S3, where the energy difference between the two configurations is 0.164 eV/2*b*. In addition, the energy difference between dipole and quadrupole stacking configurations is 0.667 eV/2*b*. Since the dislocation quadrupole is energetically more stable and reduces the contribution of configuration-dependent energy, it was chosen as a reference configuration in this work.


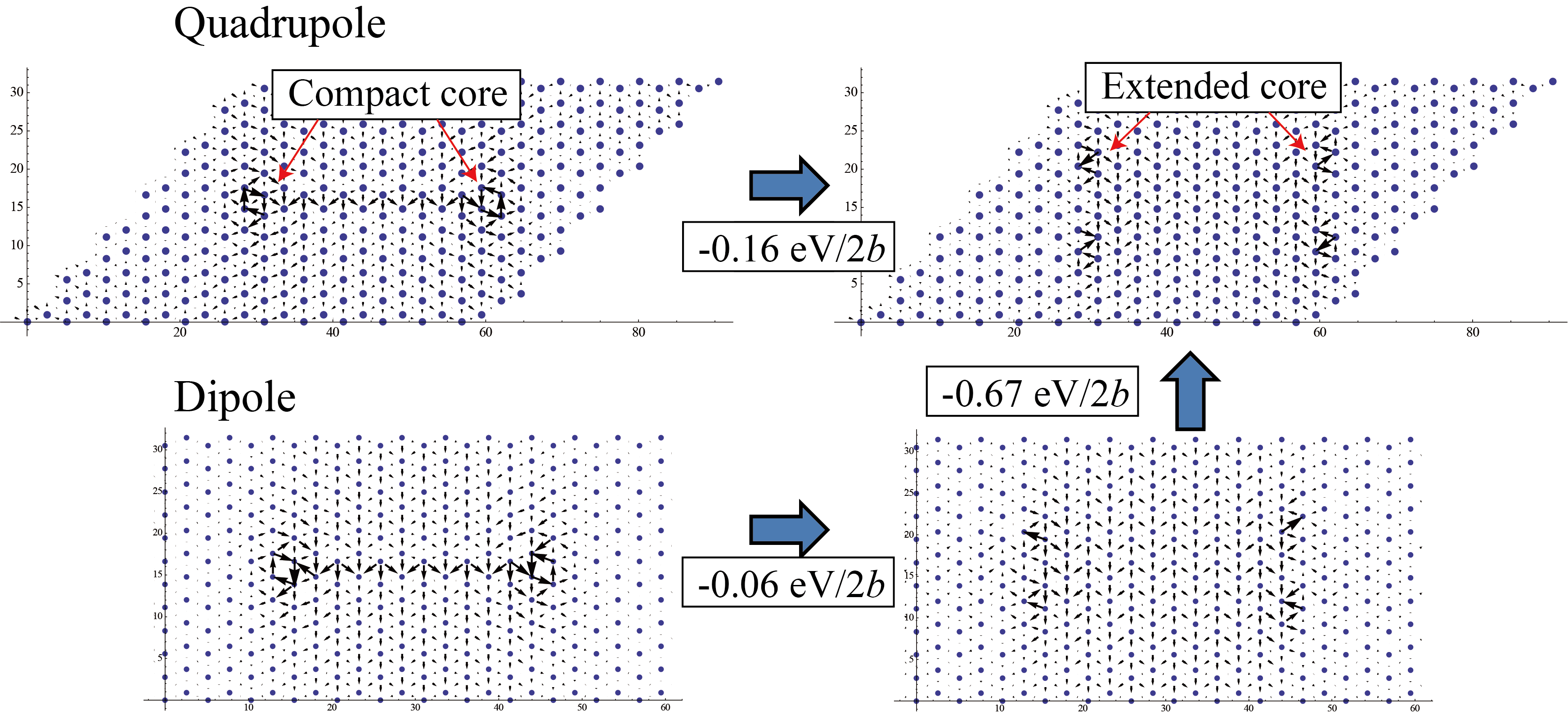


**Figure S3 | Differential displacement maps^10,11^ for dislocation dipole and quadrupole configurations.** Metastable and stable configurations are shown along with the energy differences between several configurations.

The transition state during dislocation motion is calculated using NEB with thirteen replica images (including the initial and final state). Forces for NEB calculations are converged to better than 0.01 eV/Å.

**3. Effect of Y on dislocation core structure**

Solid solutes are one of the most important agents to change the electronic structure. We have investigated the effects of solutes on the mechanical properties related to dislocation motion by constructing dislocation core structures that include one substitutional solute element. We have found that a dislocation core structure varies significantly by introducing a Y solute as shown in Fig. S4. It is interesting to note that the differential displacement maps for dislocation cores with Y show similar distributions to those for perfect dislocation cores in pure Mg. Therefore, the extended dislocation tends to shrink to form a perfect dislocation. While we showed only a typical configuration of a dislocation and a solute in the main manuscript, it would be true with various different configurations.


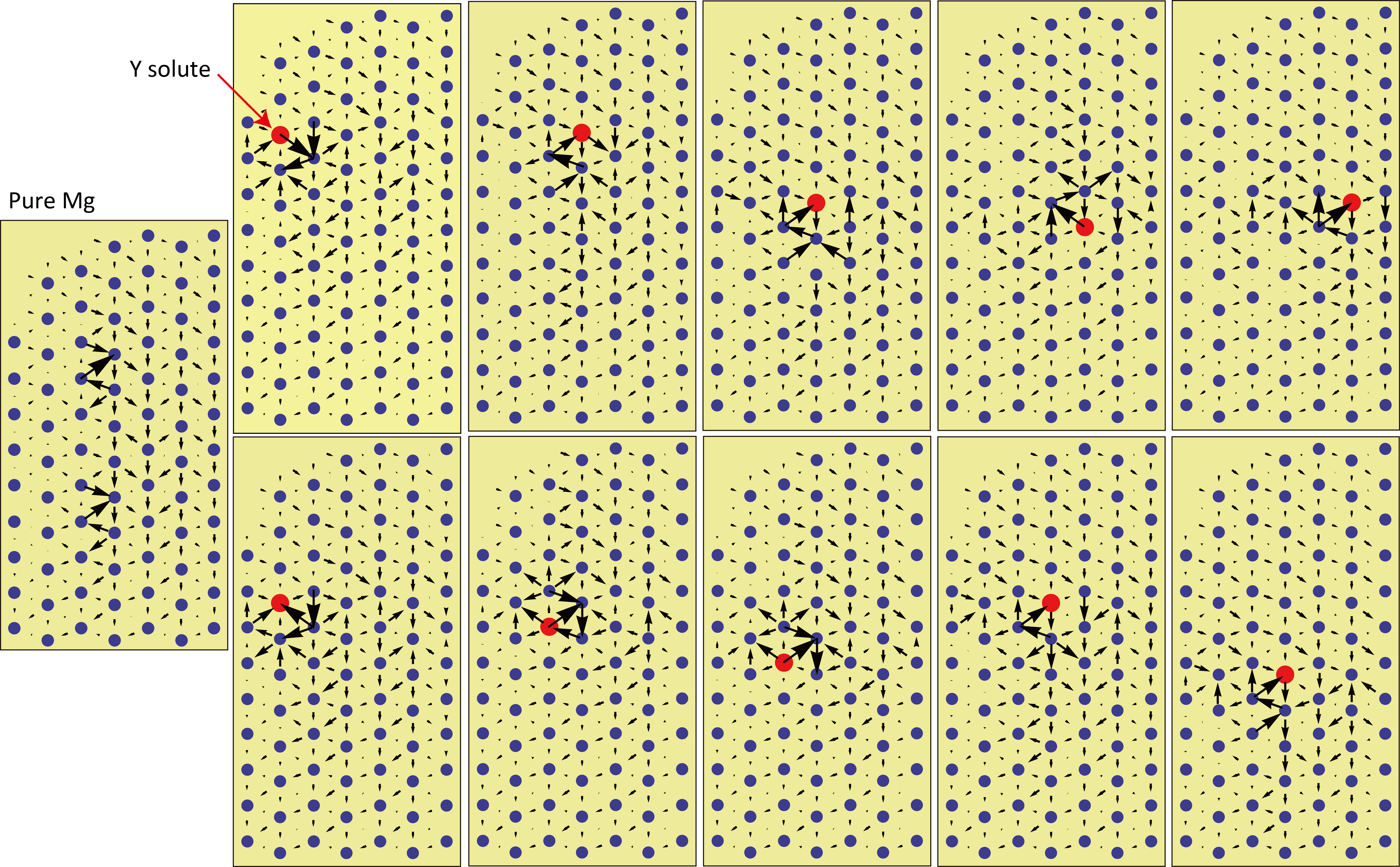


**Figure S4 | Structural change in dislocation core with Y solution.** Differential displacement maps with respect to the solute are shown. It is seen that the extended dislocations tend to shrink to form a perfect dislocation.

**4. Strengthening effect of Y solute on basal slip**

As noted in the main text, the Y solute binds strongly to the dislocation core. From Fig. 6 of the main text, we estimate that the binding energy per unit length of the line of Y atoms with the dislocation core to be *E_b_* = 0.0624 eV/Å (0.2eV/*b*). Moreover, this binding energy is formed over a short distance, $l\approx\frac{3c}{2}$ . Here, *b* is the magnitude of the Burgers vector and *c* is the lattice constant along $\left\langle0001\right\rangle$ direction.

Within obstacle-controlled glide theories, we need to know the force required to bypass the Y atom. We can estimate this force within our model by considering the change in line energy of a straight dislocation as it passes through the region influenced by the presence of the Y atom. Assuming the effects of the Y atom are felt within a spherical region with radius *l*, and that the dislocation is straight at its lowest energy configuration, we can estimate the force required to bypass the obstacle (Fig. S5). The minimum energy state for the dislocation is assumed to be straight, and passing through the lowest energy position. It is assumed that the interaction energy is varies linearly over the range of interaction. Then, the interaction energy in this state, $E_{p}$is approximated as:

$E_{p}=2\int_{0}^{l} dr E_{b}\left(\frac{r}{l}-1\right)=-E_{b} l$. (S1)

The magnitude of the pinning force, $F_{P}$, is then estimated:

$F_{p}=E_{b}$. (S2)

For basal plane dislocations in Mg, The line tension of the dislocation is approximated by $\Gamma=K_{s}b^{2}$, where the dislocation energy factor of the screw dislocation in basal plane is $K_{s}=2.93 \text{GPa}$^12^. Accordingly, by solving for , the critical depinning angle can be estimated as 159°^13-23^. This suggests that the obstacles are quite weak, a sensible result. The maximum dimensionless strength, *β_c_*, is given by $\beta_{c}=F_{p}/\left(2\Gamma\right)$. The critical stress for dislocation motion is then approximated as:

, (S3)

with $d_{Y}$ the average spacing between Y atoms on the slip plane. Assuming 1 at.-% Y, we find $d_{Y}=13.2$ Å. Substituting the values above, we find $\tau_{c}\approx90\text{MPa}$.


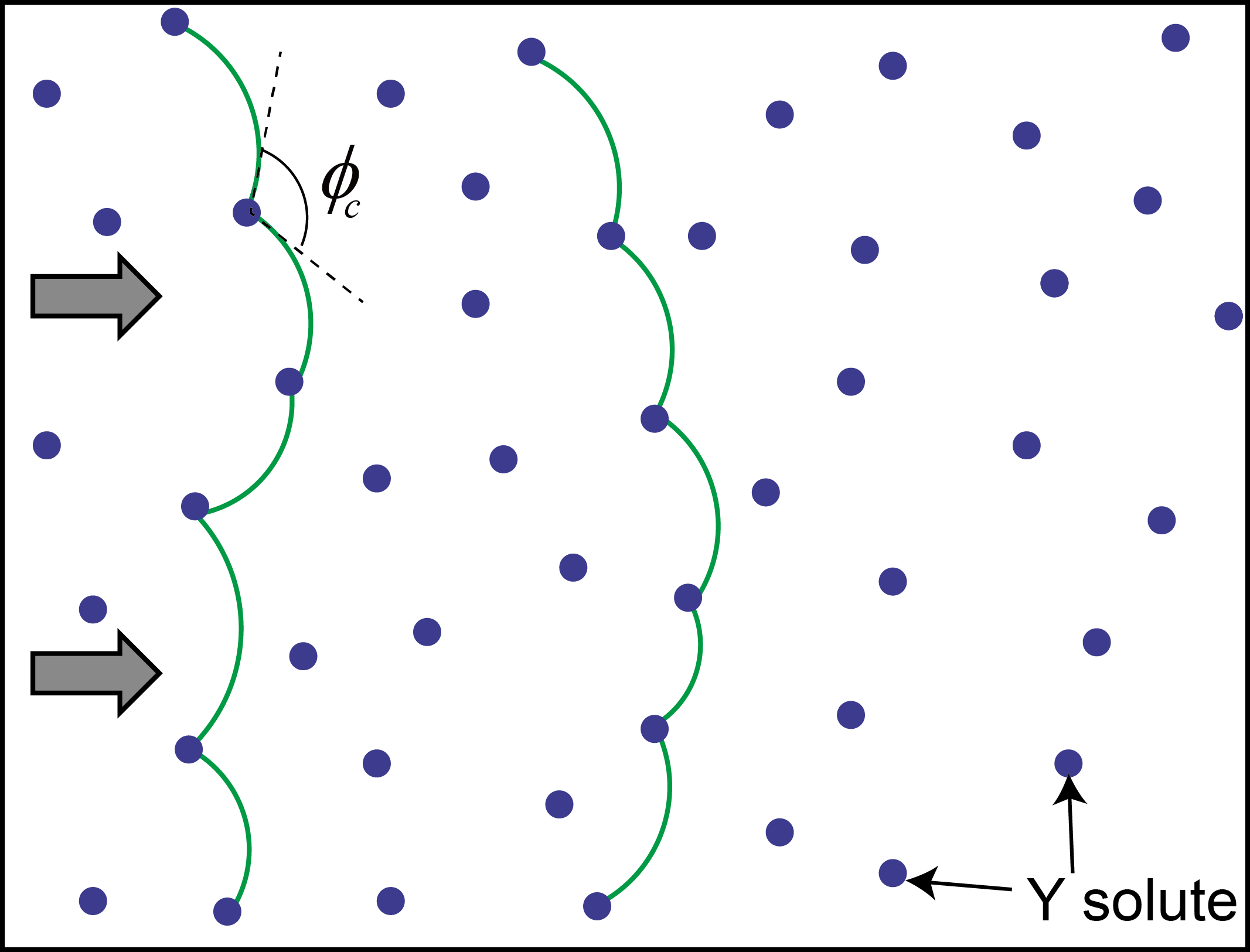


**Figure S5 | Schematic diagram for strengthening effect of distributed immobile solutes on dislocation glide.**

**References**

1. Henkelman, G., Uberuaga, B. P. and Jónsson, H. A climbing image nudged elastic band method for finding saddle points and minimum energy paths. *J. Chem. Phys.* **113**, 9901–9904 (2000).
2. Daw, M. S. Elasticity effects in electronic structure calculations with periodic boundary conditions. *Comp. Mater. Sci.* **38**, 293–297 (2006).
3. Hearmon, R. F. The elastic constants of crystals and other anisotropic materials. In: Hellwege, K. -H. & Hellwege, A. M. (Eds.), Landolt-Börnstein Tables, Group III, Vol. 11, (Springer-Verlag, Berlin, 1979).
4. Jona, F. & Marcus, P. M. Hexagonal and tetragonal states of magnesium by first principles. *Phys. Rev. B.* **66**, 094104 (2002).
5. Lehto, N. & Öberg, S. Effects of Dislocation Interactions: Application to the Period-Doubled Core of the 90 Partial in Silicon, *Phys. Rev. Lett.* **80**, 5568–5571 (1998).
6. Kresse, G. & Hafner, J. Ab initio molecular dynamics for liquid metals. *Phys. Rev. B* **47**, 558–561 (1993).
7. Kresse, G. & Furthmuller, J. Efficient iterative schemes for ab initio total-energy calculations using a plane-wave basis set. *Phys. Rev. B* **54**, 11169–11186 (1996).
8. Perdew, J. P., et al. Atoms, molecules, solids, and surfaces: Applications of the generalized gradient approximation for exchange and correlation. *Phys. Rev. B* **46**, 6671–6687 (1992).
9. Monkhorst, H. J. & Pack, J. D. Special points for Brillouin-zone integrations. *Phys. Rev. B* **13**, 5188–5192 (1976).
10. Vitek, V., Perrin, R. C. & Bowen, D. K. The core structure of ½(111) screw dislocations in b.c.c. crystals. *Philos. Mag.* **21**, 1049–1073 (1970).
11. Vitek, V. Theory of the core structures of dislocations in body-centered cubic metals. *Cryst. Latt. Def.* **5**, 1–34 (1974).
12. Tsuru, T., et al. Solution softening in magnesium alloys: the effect of solid solutions on the dislocation core structure and nonbasal slip. *J. Phys.: Condens. Matter* **25**, 022202 (2013).
13. Kocks, U. F. A statistical theory of flow stress and work-hardening. *Philos. Mag.* **13**, 541–566 (1966).
14. Kocks, U. F. Statistical treatment of penetrable obstacles. *Can. J. Phys.* **45**, 737–755 (1967).
15. Foreman, A. J. E. & Makin, M. J. Dislocation movement through random arrays of obstacle. *Philos. Mag.* **14**, 911–924 (1966).
16. Bacon, D. J., Kocks, U. F. & Scattergood, R. O. The effect of dislocation self-interaction on the Orowan stress. *Philos. Mag.* **28**, 1241–1263 (1973).
17. Hanson, K. & Morris, J. W., Jr. Estimation of the critical resolved shear stress for dislocation glide through a random mixture of distinct obstacles. *J. Appl. Phys.* **46**, 2378–2383 (1975).
18. Hanson, K. & Morris, J. W., Jr. Computer simulation of interacting dislocation motion resisted by pointlike barriers, *J. Appl. Phys.* **49**, 3266–3271 (1978).
19. Altintas, S & Morris, J.W. Computer simulation of dislocation glide—I. Comparison with statistical theories. *Acta Metall.* **34**, 801–807 (1986).
20. Altintas, S & Morris, J.W. Computer simulation of dislocation glide—II. Comparison with experiments. *Acta Metall.* **34**, 809–816 (1986).
21. Glazer, J. & Morris, J. W., Jr. The effect of the precipitate size distribution on the aging curve of order hardening alloys. *Acta Metall.* **36**, 907–915 (1988).
22. Labusch, R. Statistical theory of dislocation configurations in a random array of point obstacles. *J. Appl. Phys.* **48**, 4550–4556 (1977).
23. Chrzan, D. C., Morris, J. W., Jr., Osetsky, Y. N., Stoller, R. E. & Zinkle, S. J. What is the limit of nanoparticle strengthening? MRS Bulletin **34**, 173–177 (2009).
